# Annular Cutaneous Pili Migrans on the Scrotum: A Rare Clinical Presentation

**DOI:** 10.1002/ccr3.72369

**Published:** 2026-04-14

**Authors:** Jia Yang, Haiying Hui

**Affiliations:** ^1^ Shaanxi Provincal People's Hospital Xi'an Shaanxi China; ^2^ Xi'an Medical University Xi'an Shaanxi China; ^3^ Department of Dermatology Shaanxi Provincal People's Hospital Xi'an Shaanxi China

**Keywords:** annular, cutaneous larva Migrans, cutaneous Pili Migrans, dermoscopy, embedded hair, scrotum

## Abstract

Cutaneous Pili Migrans is a rare creeping eruption typically presenting as a linear black track on the feet. Involvement of the scrotum is extremely uncommon, and an annular morphology has not been reported. A 40‐year‐old man presented with an asymptomatic black ring‐like lesion beneath the scrotal epidermis. Dermoscopy revealed a thick circular hair shaft embedded with minimal inflammation, confirming the diagnosis. Without intervention, the lesion spontaneously resolved within one month. This first report of annular scrotal Cutaneous Pili Migrans expands the clinical spectrum and highlights the role of dermoscopy in avoiding unnecessary procedures.

## Key Clinical Message

We report the first case of annular cutaneous pili migrans on the scrotum, an extremely rare site and morphology. Dermoscopy is essential for noninvasive diagnosis, avoiding unnecessary procedures. In asymptomatic patients, conservative management without manual extraction is a valid option, as spontaneous resolution can occur.

## 1 Introduction

Cutaneous Pili Migrans, first described by Yaffee in 1957, is a rare condition characterized by a creeping eruption caused by the intradermal migration of a hair shaft [1]. It most commonly occurs on the feet and presents as a linear or wavy black track [2]. Involvement of the scrotum is exceedingly rare, and an annular presentation has never been documented. We report a unique case of annular Cutaneous Pili Migrans on the scrotum, emphasizing the diagnostic value of dermoscopy and the possibility of spontaneous resolution.

## 2 Case Presentation

**FIGURE 1:**
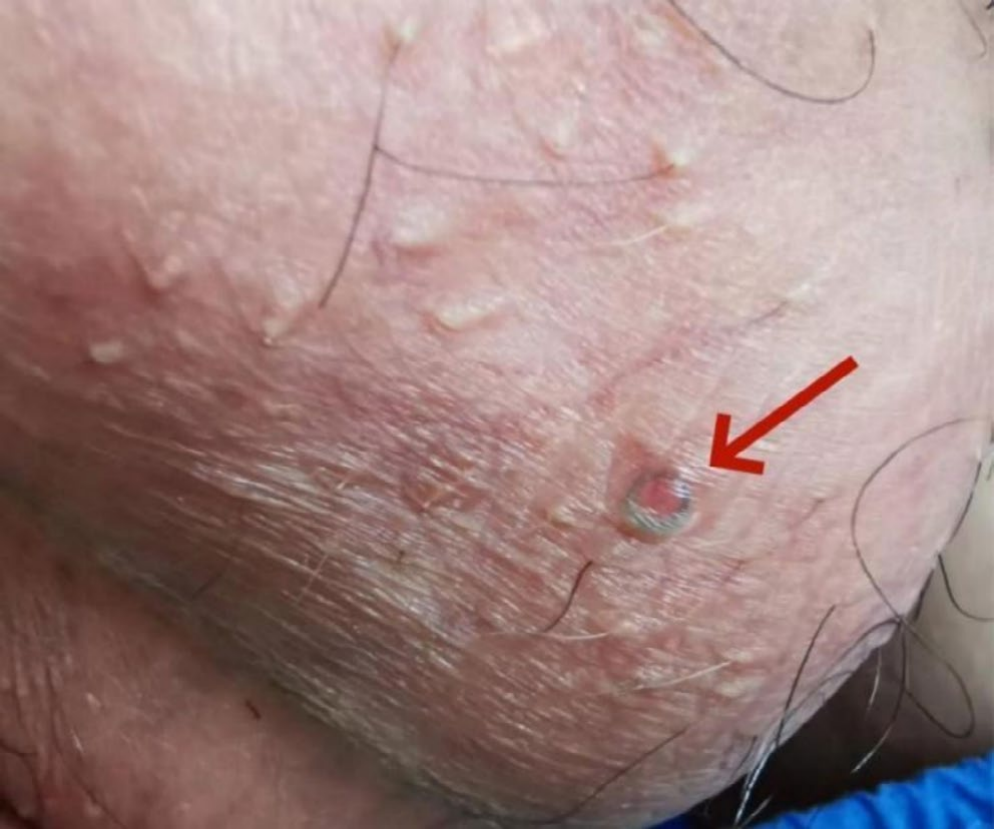
A thick, black, annular hair shaft is clearly visible beneath the scrotal epidermis, accompanied by mild central inflammation.

**FIGURE 2:**
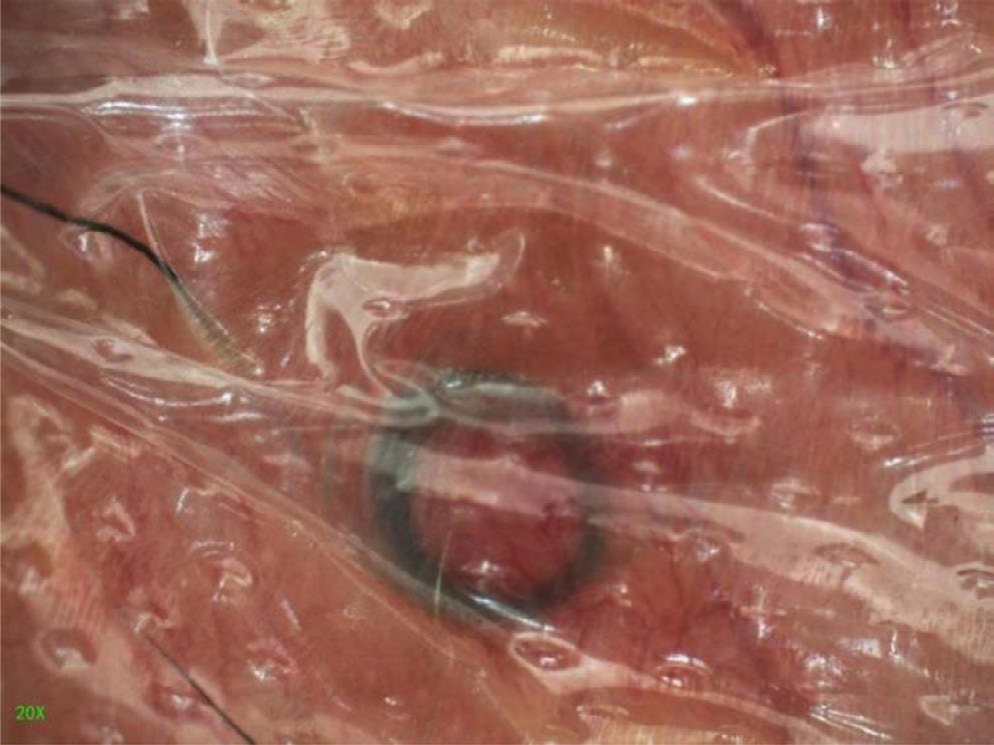
A black, annular hair shaft embedded beneath the epidermis, with no hair follicles and minimal surrounding inflammation (original magnification ×20).

A 40‐year‐old man presented with an asymptomatic black ring‐like structure beneath the scrotal skin. He denied trauma or discomfort. Physical examination showed a thick, annular hair shaft visible under the epidermis with mild central erythema (Figure 1). Dermoscopy confirmed a circular embedded hair without follicular attachment and minimal surrounding inflammation (Figure 2). Based on these findings, Cutaneous Pili Migrans was diagnosed. Given the absence of symptoms, conservative management was chosen. At one‐month follow‐up, the lesion had completely resolved without recurrence.

## 3 Discussion

Cutaneous Pili Migrans typically manifests as a linear black lesion on the foot, with reported sites including the ankle, toe, palm, trunk, and head [2]. Scrotal involvement has not been previously described. We report the first case with an annular configuration on the scrotum. This morphology may result from the naturally curly nature of scrotal hair, which coils as it grows inward.

Differential diagnosis includes cutaneous larva migrans (CLM), interdigital pilonidal sinus, and pseudofolliculitis barbae [3]. CLM typically presents with serpiginous tracks, intense pruritus, and eosinophilic infiltration, whereas Cutaneous Pili Migrans exhibits a distinct black line with minimal inflammation and may be asymptomatic [3]. In our case, dermoscopy excluded larvae, and the lack of pruritus and spontaneous resolution further distinguished it from CLM.

Dermoscopy is essential for noninvasive diagnosis, avoiding unnecessary invasive treatment. This case also demonstrates that asymptomatic lesions may resolve spontaneously, supporting a conservative approach. Clinicians should be aware of this atypical presentation to prevent misdiagnosis and overtreatment.

## Author Contributions

**Jia Yang:** writing – original draft, writing – review and editing. **Haiying Hui:** conceptualization, investigation, methodology, resources, project administration.

## Funding

The authors have nothing to report.

## Consent

Written informed consent was obtained from the patient to publish this report in accordance with the journal's patient consent policy.

## Conflicts of Interest

The authors declare no conflicts of interest.

## Data Availability

The data will be made available upon request after the publication of this study.
